# Supplementary material for: OpenMM 7: Rapid development of high performance algorithms for molecular dynamics
Source: PLoS Comput Biol. 2017 Jul 26;13(7):e1005659. doi: 10.1371/journal.pcbi.1005659 (PMC5549999; doi:10.1371/journal.pcbi.1005659)
Supplement: S1 Source Code — (ZIP) [file pcbi.1005659.s002.zip › OpenMM-7.0.1-Source/docs/api-python/generated/simtk.openmm.openmm.HarmonicBondForce.html]

HarmonicBondForce — OpenMM 7.0.1.dev-5e86c4f documentation


# HarmonicBondForce¶

*class* `simtk.openmm.openmm.``HarmonicBondForce`(*\*args*)¶
:   This class implements an interaction between pairs of particles that varies harmonically with the distance between them. To use it, create a HarmonicBondForce object then call addBond() once for each bond. After a bond has been added, you can modify its force field parameters by calling setBondParameters(). This will have no effect on Contexts that already exist unless you call updateParametersInContext().

    `__init__`(*self*) → HarmonicBondForce¶
    :   \_\_init\_\_(self, other) -> HarmonicBondForce

        Create a HarmonicBondForce.

    Methods

    |  |  |
    | --- | --- |
    | `__init__`((self) -> HarmonicBondForce) | \_\_init\_\_(self, other) -> HarmonicBondForce |
    | `addBond`((self, particle1, particle2, length, ...) | Add a bond term to the force field. |
    | `getBondParameters`(self, index) | Get the force field parameters for a bond term. |
    | `getForceGroup`((self) -> int) | Get the force group this Force belongs to. |
    | `getNumBonds`((self) -> int) | Get the number of harmonic bond stretch terms in the potential function |
    | `setBondParameters`(self, index, particle1, ...) | Set the force field parameters for a bond term. |
    | `setForceGroup`(self, group) | Set the force group this Force belongs to. |
    | `updateParametersInContext`(self, context) | Update the per-bond parameters in a Context to match those stored in this Force object. |
    | `usesPeriodicBoundaryConditions`((self) -> bool) | Returns whether or not this force makes use of periodic boundary conditions. |

    `getNumBonds`(*self*) → int¶
    :   Get the number of harmonic bond stretch terms in the potential function

    `addBond`(*self*, *particle1*, *particle2*, *length*, *k*) → int¶
    :   Add a bond term to the force field.

        |  |  |
        | --- | --- |
        | Parameters: | - **particle1** (*int*) – the index of the first particle connected by the bond - **particle2** (*int*) – the index of the second particle connected by the bond - **length** (*double*) – the equilibrium length of the bond, measured in nm - **k** (*double*) – the harmonic force constant for the bond, measured in kJ/mol/nm^2 |
        | Returns: | the index of the bond that was added |
        | Return type: | int |

    `getBondParameters`(*self*, *index*)¶
    :   Get the force field parameters for a bond term.

        |  |  |
        | --- | --- |
        | Parameters: | **index** (*int*) – the index of the bond for which to get parameters |
        | Returns: | - **particle1** (*int*) –   the index of the first particle connected by the bond - **particle2** (*int*) –   the index of the second particle connected by the bond - **length** (*double*) –   the equilibrium length of the bond, measured in nm - **k** (*double*) –   the harmonic force constant for the bond, measured in kJ/mol/nm^2 |

    `setBondParameters`(*self*, *index*, *particle1*, *particle2*, *length*, *k*)¶
    :   Set the force field parameters for a bond term.

        |  |  |
        | --- | --- |
        | Parameters: | - **index** (*int*) – the index of the bond for which to set parameters - **particle1** (*int*) – the index of the first particle connected by the bond - **particle2** (*int*) – the index of the second particle connected by the bond - **length** (*double*) – the equilibrium length of the bond, measured in nm - **k** (*double*) – the harmonic force constant for the bond, measured in kJ/mol/nm^2 |

    `updateParametersInContext`(*self*, *context*)¶
    :   Update the per-bond parameters in a Context to match those stored in this Force object. This method provides an efficient method to update certain parameters in an existing Context without needing to reinitialize it. Simply call setBondParameters() to modify this object’s parameters, then call updateParametersInContext() to copy them over to the Context.

        The only information this method updates is the values of per-bond parameters. The set of particles involved in a bond cannot be changed, nor can new bonds be added.

    `usesPeriodicBoundaryConditions`(*self*) → bool¶
    :   Returns whether or not this force makes use of periodic boundary conditions.

        |  |  |
        | --- | --- |
        | Returns: | true if force uses PBC and false otherwise |
        | Return type: | bool |

    `__delattr__`¶
    :   x.\_\_delattr\_\_(‘name’) <==> del x.name

    `__format__`()¶
    :   default object formatter

    `__getattribute__`¶
    :   x.\_\_getattribute\_\_(‘name’) <==> x.name

    `__hash__`¶

    `__reduce__`()¶
    :   helper for pickle

    `__reduce_ex__`()¶
    :   helper for pickle

    `__sizeof__`() → int¶
    :   size of object in memory, in bytes

    `__str__`¶

    `getForceGroup`(*self*) → int¶
    :   Get the force group this Force belongs to.

    `setForceGroup`(*self*, *group*)¶
    :   Set the force group this Force belongs to.

        |  |  |
        | --- | --- |
        | Parameters: | **group** (*int*) – the group index. Legal values are between 0 and 31 (inclusive). |

High performance molecular simulation on GPUs


### Navigation

- Application Layer
- Library Layer
  - Core Objects
  - Forces
  - Integrators
  - Extras

©2015, Stanford University and the Authors.
|
Powered by Sphinx 1.3.1
& Alabaster 0.7.7
|
Page source
